# Identification and validation of stemness-associated hub genes in cervical cancer: a bioinformatics and experimental study

**DOI:** 10.1186/s12957-026-04472-7

**Published:** 2026-06-27

**Authors:** Shama Prasada Kabekkodu, Alfa Florence Rodrigues, Pratheeksha Hebbar, Samatha Bhat

**Affiliations:** 1 Department of Cell and Molecular Biology, Manipal School of Life Sciences, Manipal Academy of Higher Education, Manipal, India; 2 Department of Biotherapeutics Research, Manipal Academy of Higher Education, Manipal, India

**Keywords:** Cervical cancer, stem cells, differential expression, hub genes, prognostic marker, immune infiltration

## Abstract

Cancer stem cells (CSCs) are a subpopulation with self-renewal and differentiation capacity that drive the progression, recurrence, and therapeutic resistance of patients with cervical cancer (CC). However, the complete set of genes that maintain stemness in CC remains incompletely defined. We aimed to identify key stemness-related genes and evaluate their prognostic utility, immune associations, and drug sensitivity. Through literature mining and CellMarker 2.0, we identified 1345 stemness-associated genes that overlapped with differentially expressed genes (DEGs) from the TCGA-CESC dataset (log2FC > 2, *p* < 0.05), yielding 216 stemness-related DEGs. A protein-protein interaction network (STRING) and CytoHubba (MCC algorithm) revealed ten hub genes (HGs): *CCNB1*,* CCNA2*,* BUB1B*,* UBE2C*,* KIF11*,* CCNB2*,* KIF23*,* CDC20*,* CDC6*, and *FOXM1*. Gene ontology and KEGG analyses revealed predominant enrichment in cell cycle progression. Cox regression and Kaplan‒Meier analyses identified *BUB1B*,* CCNA2*,* CDC20*,* FOXM1*,* and KIF23* as risk factors for poor overall survival, with *KIF11* emerging as an independent prognostic factor. HGs overexpression significantly correlated with altered infiltration of 15 immune cell types, including negative associations with CD8 + T and NK cells. We identified 661 unique drugs/chemicals targeting these HGs, including FDA-approved repurposed agents. Experimental validation via RT‒PCR confirmed significant overexpression of *FOXM1* and *KIF11* in CC tissues and cell lines compared with normal samples. These stemness-associated HGs, particularly *FOXM1* and *KIF11*, may serve as potential prognostic biomarkers and therapeutic targets, warranting further investigation of stemness-driven CC progression.

**Supplementary Information:**

The online version contains supplementary material available at https://doi.org/10.1186/s12957-026-04472-7.

## Introduction

Cervical cancer (CC) is the fourth most common gynecological malignancy worldwide. CC contributes approximately 661,021 new cases and 348,189 deaths in 2022 [1]. Low- and middle-income countries have the highest incidence and mortality rates of CC [1]. Persistent infection with high-risk human papillomavirus (HPV) is the major risk factor for CC. Vaccination against carcinogenic HPV types, Pap testing, and treatment of precancerous conditions have significantly reduced the incidence and mortality of CC worldwide [2]. Despite improvements in diagnosis, prognosis, and treatment, the mortality rate due to CC remains among the top four worldwide. Most CC patients are diagnosed when the disease progresses to an advanced stage. CCs detected at an advanced stage are difficult to treat and are associated with treatment failure and a high rate of recurrence [2]. Therefore, identifying molecular markers that can improve the clinical management of CC is important.

Several studies have demonstrated that cancer stem cells (CSCs) may play a critical role in CC progression by promoting metastasis, recurrence, and therapeutic resistance [3]. Recent studies have shed light on additional molecular mechanisms and therapeutic strategies to address the persistent challenges in CC management. For example, the circular RNA hsa_circ_0000021 promotes CC progression by sponging miR-3940-3p and upregulating KPNA2 expression [4]. Furthermore, natural compounds such as extracts from *Abelmoschus esculentus* (okra) have been shown to have selective anticancer effects on CC cell lines but have fewer effects on normal cells [5]. Similarly, the combination of myricetin and epigallocatechin gallate (EGCG) demonstrated synergistic antiproliferative effects in SiHa and ME180 cells [6]. These findings highlight the ongoing need for novel molecular biomarkers and targeted therapies to overcome resistance driven by CSCs. Moreover, previous studies have reported various genes (*ABCG2*,* MSI1*,* ALDH1*,* CK17*,* CD44*,* CD133*,* PROM1*,* ITGA6*,* POU5F1*,* OCT3*) as markers with the potential to induce stem-like properties in CC cell lines [7]. The interaction of CSCs with immune cells and the tumor microenvironment (TME) is proposed to be critical for CC progression. Cervical cancer stem cell populations are highly resistant to conventional treatment regimens, leading to treatment failure. For example, OCT4 expression is positively correlated with radioresistance in squamous cell carcinoma of the cervix [8]. Similarly, the side population isolated from HeLa cells presented high expression of CD133 and chemo-/radioresistance [9]. SOX2 and CD49f contribute to stem cell-like properties, such as enhanced tumorigenicity, self-renewal, and increased expression of epithelial-to-mesenchymal transition (EMT) and stem cell-specific markers [10]. Thus, given its capacity to drive carcinogenesis, the CSC population is a unique target for improved therapeutic outcomes. Hence, identifying, characterizing and targeting various stem cell-related markers and associated pathways may provide insights that could support the development of improved therapeutic strategies for CC.

Research has demonstrated that stemness-related genes can be accurately identified via in silico investigations of freely available genomics and transcriptomics datasets, with the potential to identify both diagnostic and prognostic markers [11, 12]. Bioinformatics approaches have been successfully employed to identify stemness-related genes and hub genes (HGs) in multiple cancers. For example, integrated bioinformatics approaches combining the mRNA stemness index (mRNAsi) with weighted gene coexpression network analysis (WGCNA) have been used to identify stemness-associated HGs in kidney renal clear cell carcinoma [13], lung adenocarcinoma [14], and breast cancer [15]. However, studies specifically focusing on stemness-related HGs in CC remain relatively limited. A previous study investigated stemness-related signatures in cervical squamous cell carcinoma and endocervical adenocarcinoma via a combined bioinformatics approach [16]. The study was conducted using mRNAsi along with WGCNA and protein‒protein interaction networks on the TCGA-CESC dataset to study stemness-associated HGs primarily enriched in cell proliferation and cell cycle pathways. While their study provided valuable insights into stemness signatures in the two major histological subtypes, our study extends beyond their work in several critical aspects: (i) we analyzed the complete TCGA-CESC dataset, including all histological subtypes; (ii) we integrated drug sensitivity analysis from both the CTRP and GDSC databases, identifying 661 unique compounds; (iii) we performed immune infiltration analysis across 24 immune cell types; (iv) we provided experimental validation of two HGs (*FOXM1* and *KIF11*) in clinical tissue samples and cell lines; and (v) we used multiple algorithms (MCC, degree, and EPC) for HGs identification to ensure robustness. In summary, our bioinformatics analysis revealed potential stemness-related genes and their clinical relevance in CC. Targeting these genes may influence CSC-associated pathways involved in CC progression.

## Materials and methodology

### Literature search

A comprehensive literature search was conducted to identify studies on stemness-associated genes in CC. Electronic databases (Embase, PubMed, Web of Science, and Scopus) were searched from inception to January 2025. The search strategy combined keywords and Boolean operators: (“stemness” OR “cancer stem cells” OR “self-renewal” OR “stemness markers”) AND (“cervical cancer” OR “cervical carcinoma”) AND (“gene expression” OR “survival” OR “prognostic biomarkers”). A total of 1,847 articles were screened by title and abstract. After removing duplicates (*n* = 423) and irrelevant studies (*n* = 1,289), 135 full-text articles were reviewed for gene extraction. Among these genes, 1,345 unique stemness-associated genes were identified.

### Data acquisition and analysis

The study included RNA-seq data from 304 tumor samples from The Cancer Genome Atlas- Cervical Squamous Cell Carcinoma and Endocervical Adenocarcinoma (TCGA-CESC) and 22 normal samples from TCGA-CESC and the Genotype-Tissue Expression (GTEx) portal V11 [17]. Clinical covariates such as tumor stage and patient age were not included in the analysis because of incomplete annotation or unavailability of the data in the selected datasets. CellMarker 2.0 is a database that provides manually curated and experimentally supported markers from different tissues from humans and mice [18]. CellMarker 2.0 integrates information for 26,915 markers, 2578 different cell types, and 656 tissues that are manually curated from more than 24,500 published studies. We downloaded the data from the CellMarker 2.0 database via the “All cell markers” module available under the download tab. The data were exported to Microsoft Excel, and genes related to “Stem Cells” were retrieved via the “Sort and Filter” option. We also conducted a literature search using the term “stem cells” in PubMed and Google Scholar to identify additional genes related to stemness. Transcriptome Alterations in the CanCer Omnibus V1 is a webserver designed to integrate transcriptomics data, pathway alterations and clinical outcomes (TACCO) from TCGA datasets [19]. We downloaded the list of DEGs (log2-fold change, *P* < 0.05) from the TCGA-CESC transcriptomics dataset via the TACCO interface. All subsequent analyses were carried out using common genes between CellMarker 2.0 and genes identified by a literature search with DEGs in the TCGA-CESC dataset as identified by Venny 2.0.

### Identifying the differentially expressed genes (DEGs)

We identified DEGs between CC and normal tissues by applying the following parameters: (i) log2-fold change| >2 and (ii) adjusted < 0.05 via the TACCO webserver. To compute differential expression, we used the normalized mean transcript per million (TPM) of normal and tumor samples. The Wilcoxon test was performed to test whether there was a difference between normal and tumor samples.

### Protein‒protein interaction network (PPIN) and identification of hub genes

We used STRING V 12.0 to construct the PPIN [20]. We included evidence from text mining, experiments, databases, co-expression, neighborhoods, gene fusion, co-occurrence and the highest confidence score of 0.9 for generating the PPIN. The top ten highly connected genes or HGs were identified by screening the PPIN via the maximal clique centrality (MCC) algorithm in the CytoHubba tool V0.1 and visualized via Cytoscape 3.10.2 [20, 21].

### Immunohistochemistry (IHC) analysis using the Human Protein Atlas (HPA)

We performed an in silico evaluation of HGs expression in normal (*n* = 2) and CC tissue (*n* = 12) using IHC data available from the HPA database. The IHC staining intensity was already reviewed and reported as high, medium, low, and not detected according to the HPA scoring system in the HPA database. Normal and tumor samples were compared for HGs expression frequencies and reported.

### Gene Ontology (GO) and Kyoto Encyclopedia of Genes and Genomes (KEGG) term enrichment analyses

ShinyGO 0.85 is a graphical gene-term enrichment tool applied to identify the GO and pathway terms enriched in each set of genes [22]. Using the ShinyGO 0.85 webserver, GO and KEGG (Release 117) term enrichment analyses were carried out with an FDR cutoff < 0.05.

### Evaluating the differential expression, genetic alterations and DNA methylation changes of HGs

The expression of HGs in normal (*n* = 22) and tumor (*n* = 304) samples was evaluated via the OncoDB V2.0 webserver [23]. We assessed the genomic changes in HGs in the TCGA-CESC cohort via the cBioPortal tool. The differences in promoter DNA methylation between normal and tumor samples in the TCGA-CESC datasets were determined via the UALCAN online tool [24]. HGs expression was cross-validated via an independent CC dataset (GSE9750) [25].

### Assessment of the clinical value of hub genes

To evaluate the prognostic utility of HGs expression, survival analysis was conducted with the TCGA-CESC dataset via the Tumor Online Prognostic Analyses Platform (ToPP V1.2) webserver [26]. A prognostic model for overall survival was constructed on the basis of the expression of all the HGs. The log-rank test and Kaplan‒Meier (K‒M) analysis were carried out to determine the survival value. We created a prognostic model for overall survival (OS) by including tumor grade as a clinical covariate. Multivariate analysis included 10 HGs, the best cutoff, and histological grade as clinical covariates. The differences in survival were estimated using K-M survival analysis to estimate the hazard ratio. A P value less than 0.05 was considered to indicate statistical significance.

### Stemness signature enrichment analysis

We used the StemChecker webserver V1.2.1 to explore the presence of stemness signature enrichment analysis [27]. The genes related to stemness among the DEGs were used as inputs in the StemChecker webserver to identify the cell type, stemness signatures and transcription factors associated with the DEGs. The significance is calculated via a hypergeometric test. The program uses Bonferroni corrections to calculate the adjusted p value.

### Immune infiltration and cancer hallmark enrichment analysis

We used the Gene Set Cancer Analysis (GSCA V2.0) webserver to determine the associations between HGs expression and immune infiltration [28]. GSCA is an online platform that integrates information related to genomics, drugs and immunogenic gene enrichment analysis. We used the “immune” module of the GSCA to determine the association between HGs expression and immune infiltration. Immune enrichment analysis of 24 different immune cells was performed via the ImmuCellAI algorithm in the GSCA. We selected the “enrichment plot” option for cancer hallmarks to identify overrepresented cancer hallmarks by comparing the HGs with an integrated cancer hallmark gene set consisting of 6763 genes [29].

### Gene set variation analysis (GSVA)

The GSVA score for the HGs was obtained using the Gene Set Cancer Analysis (GSCA) online tool (https://guolab.wchscu.cn/GSCA/#/). We investigated the associations between the expression of HGs and clinical outcomes using GSVA. The analysis included 306 tumor samples and 3 normal samples from the TCGA-CESC dataset. The GSVA score ranged from − 1 to + 1, with higher scores indicating greater enrichment. The GSVA score was calculated on the basis of the mRNA expression of the set of genes. GSVA scores were also compared across clinical stages (I–IV) to evaluate the association between stemness enrichment and disease progression.

### Drug‒gene interaction analysis

We used the GSCA online web server to identify the associations between HGs and potential small molecules and drugs [28]. The GSCA combines more than 10,000 genomic data and 750 small molecules to predict potential drug resistance and sensitivity on the basis of genes generated from the TCGA. The GSCA uses OMICS data from the TCGA and small molecules and drugs from the Cancer Therapeutics Response Portal (CTRP) V2.0 and the Genomics of Drug Sensitivity in Cancer (GDSC) Project. The chemical-HGs interactions were built via the STITCH V5.0 web server with the highest confidence level of 0.9 and default interaction parameters [30].

### Semiquantitative reverse transcription‒PCR

SiHa, C33A, and HeLa cells were maintained according to American Type Culture Collection guidelines in DMEM supplemented with 10% fetal bovine serum (FBS) (HiMedia, Mumbai, India). Ten cervical biopsy samples from healthy individuals and patients who were diagnosed with cervical cancer at Kasturba Medical College, Manipal, India, were included in the study. All participants provided informed consent in compliance with the Kasturba Hospital ethical committee approval (IEC763/2016). The clinical status of the samples was confirmed via histopathological examination. RNA was isolated from normal and CC tissue biopsy samples and cell lines via the TRIzol method. Total RNA was extracted via RDP trio reagent (HiMedia, Mumbai, India), and cDNA was synthesized via PhiScript 1st Strand cDNA Synthesis Mastermix (5X) (Dx/Dt, Bangalore, India) according to the manufacturer’s instructions and used for RT‒PCR. The sequences of the primers used for RT‒PCR are listed in Table 1. Relative gene expression was measured via ImageJ software, with *ACTB* (β-actin) serving as an internal control. The experiments were performed in duplicate and repeated three times.

**Table 1:** List of primers used for gene expression analysis of the 2 hub genes

Primer name	Primer sequence (5′–3′)	Product size	Annealing temperature
FOXM1-RT‒PCR-F FOXM1-RT‒PCR-R	TCACAGCAGAAACGACCGAA TCACCGGGAACTGGATAGGT	139 bp	60 °C
ACTB-RT‒PCR-F ACTB-RT‒PCR-R	GACGACATGGAGAAAATCTG ATGATCTGGGTCATCTTCTC	131 bp	58 °C
KIF11-RT‒PCR-F KIF11-RT‒PCR-R	GATGGACGTAAGGCAGCTCA TGTGGTGTCGTACCTGTTGG	185 bp	62 °C

## Results

### Data retrieval and differential expression analysis 

**Fig. 1:**
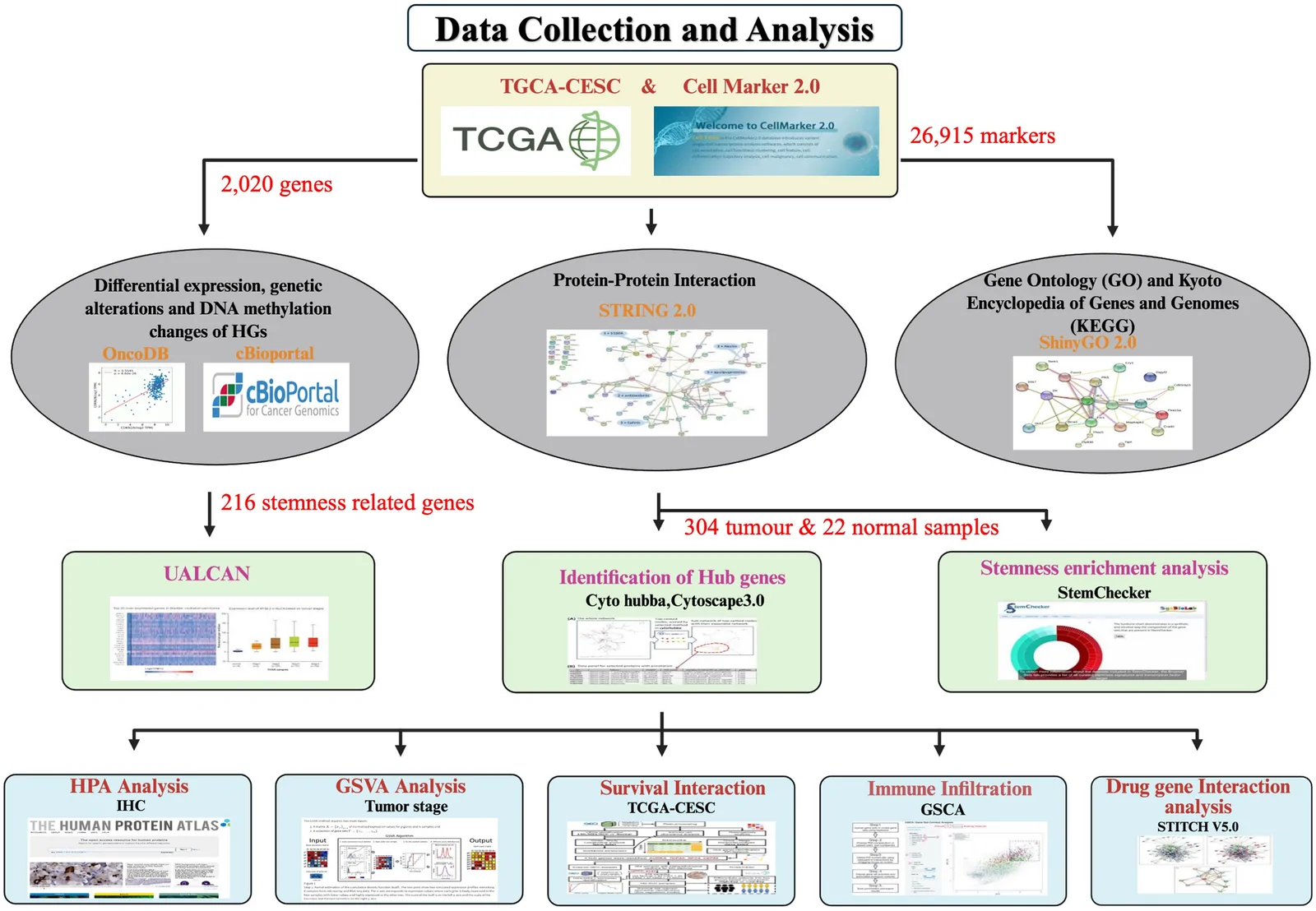
Schematic diagram representing the workflow used in the present study

**Fig. 2:**
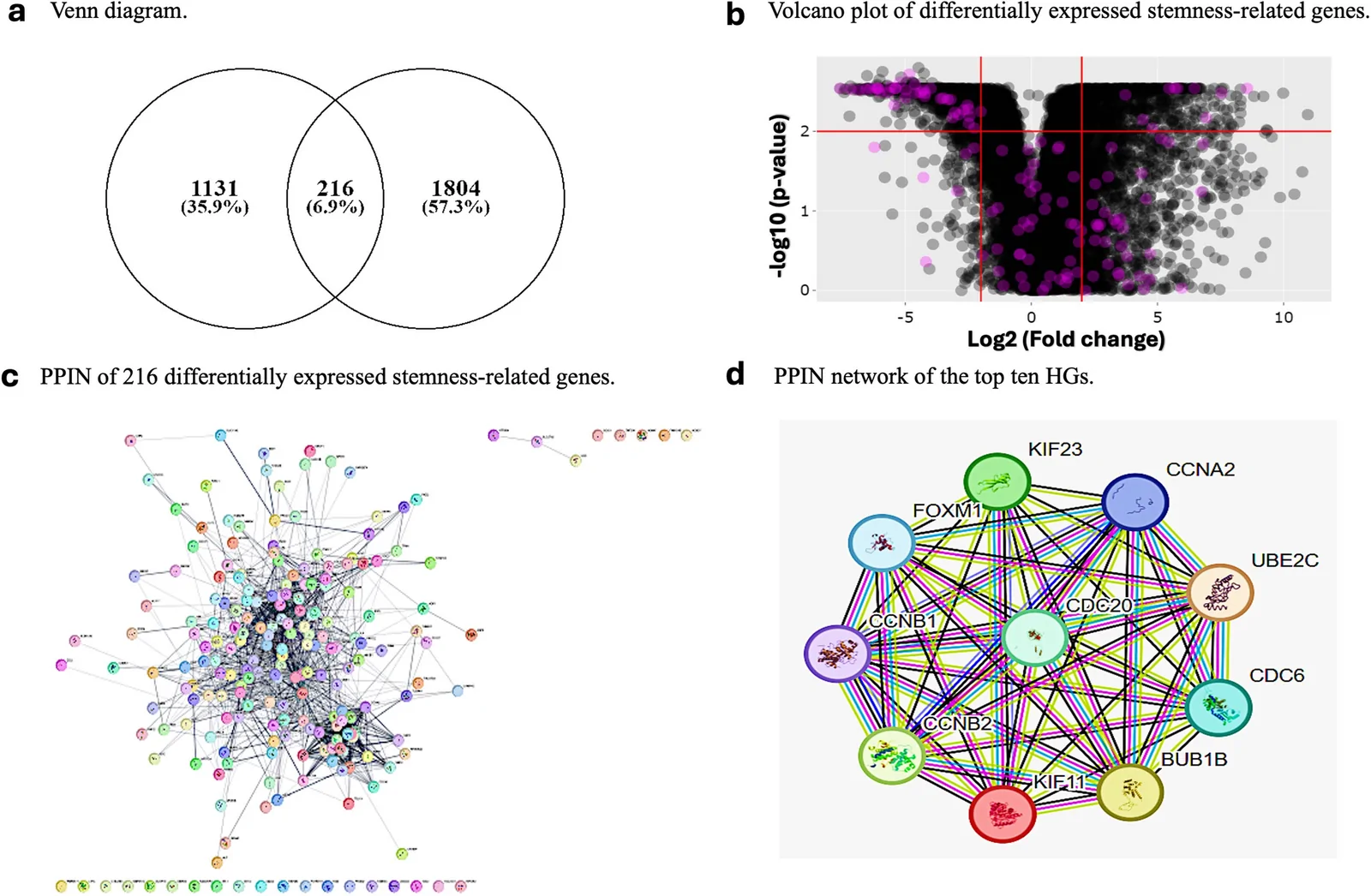
Identification of stemness-related genes in CC. **a** Venn diagram representing 216 stemness-related genes that are significantly differentially expressed between tumor and normal samples. **b** Volcano plot showing the 216 genes (pink dots) that are significantly differentially expressed between normal and tumor samples according to TCGA-CESC analysis via the TACCO tool. **c** PPIN of 216 differentially expressed stemness-related genes generated via STRING V12.0. **d** PPIN network of the top ten HGs according to cytoHubba analysis

Fig. 1 shows the overall approach used to identify stemness-related genes in CC. Analysis of the TCGA-CESC dataset via the TACCO webserver revealed that 2020 genes (upregulated: 802 genes; downregulated: 1218 genes) were differentially expressed, with a log2-fold change and *P* < 0.05 (Supplementary Table S1). A literature search and CellMarker 2.0 analysis revealed 1345 genes associated with stemness (Supplementary Table S2). Overlapping analysis between stemness-related genes and DEGs in the TCGA-CESC dataset revealed 216 differentially expressed stemness-related genes (Fig. 2a and b, Supplementary Table S3).

### Stemness-related PPIN and HGs

Relationships between the 216 differentially expressed stemness-related genes were identified via STRING V12.0 (Fig. 2c). The PPIN of stemness-related genes included 216 nodes and 209 edges with a PPI enrichment of *P* < 1.0e-16. We used the MCC algorithm to evaluate the relationships among the PPINs. Our analysis via cytoHubba revealed *CCNB1*,* CCNA2*,* BUB1B*,* UBE2C*,* KIF11*,* CCNB2*, *KIF23*,* CDC20*,* CDC6*, and *FOXM1* as the top 10 highly connected HGs (Fig. 2d).

### Testing for DNA methylation and genetic alterations in HGs

We next performed DNA methylation and mutation analysis of HGs in the TCGA-CESC dataset. Among the ten HGs, *CCNB1* and *CCNA2* were significantly differentially methylated between tumor and normal samples (Supplementary Fig. S1). Using cBioportal, we evaluated the genomic changes in HGs in the TCGA-CESC dataset. Missense mutations, splice mutations, truncating mutations, amplifications, and deletions have been reported for few genes. Among the ten HGs, *KIF11* presented the greatest number of alterations (Supplementary Fig. S2).

### Functional enrichment analysis

**Fig. 3:**
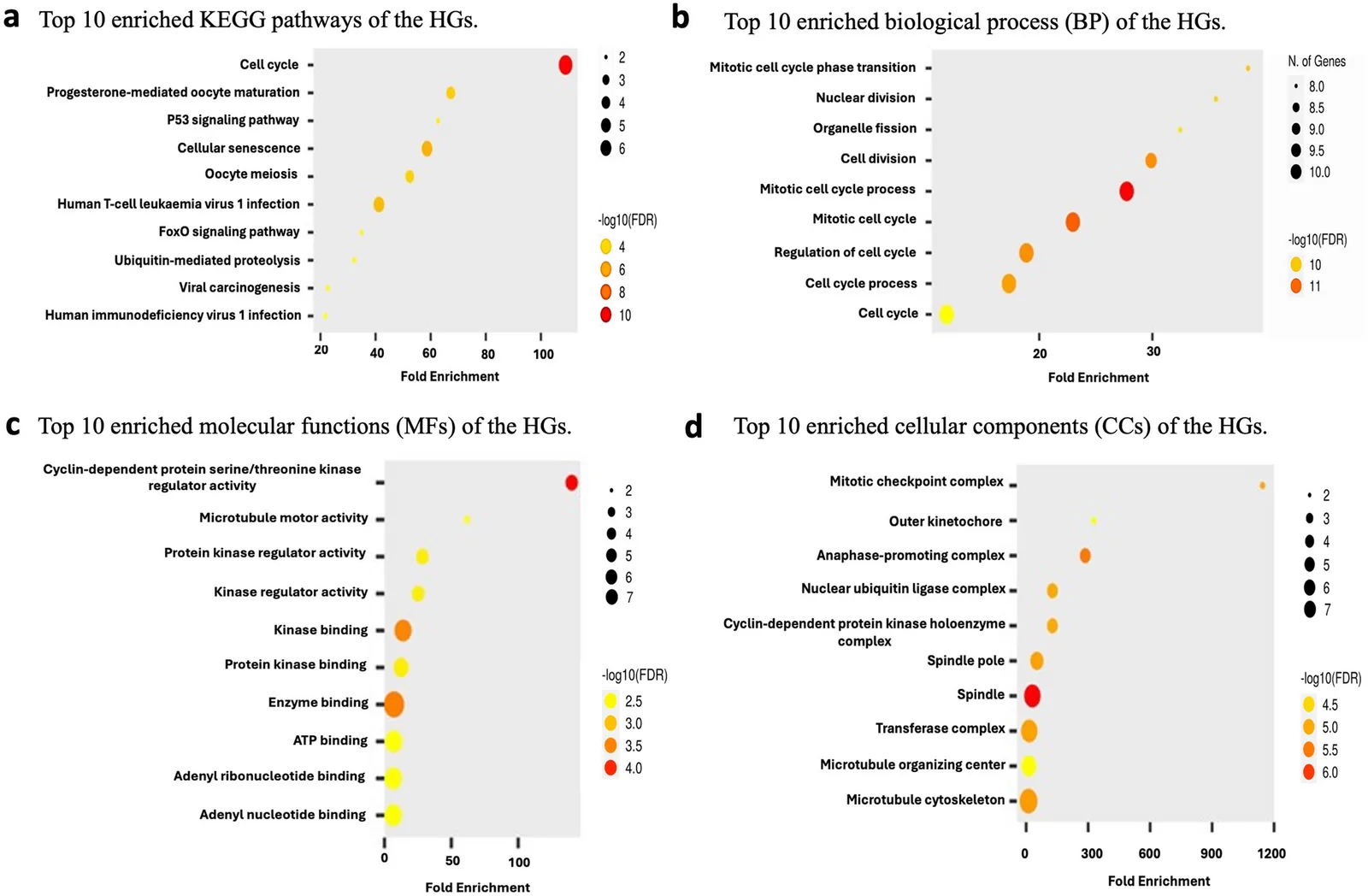
Pathway and GO term enrichment analysis of HGs. **a** The top ten enriched KEGG pathways are shown. **b** The ten 10 enriched biological process (BP) terms. **c** The top ten enriched molecular functions (MFs). **d** The top ten enriched cellular components (CCs) are shown. The analysis was performed in ShinyGO 0.80

The top ten enriched GO terms and pathways are shown in Fig. 3. The enriched biological processes, molecular functions, and cellular components related to HGs expression are shown in Fig. 3a, b and c, and 3d, respectively. Collectively, the results of the GO analysis indicate that HGs are enriched in processes related to cell division and cell cycle regulation. KEGG-based pathway enrichment analysis of the HGs revealed that the cell cycle, progesterone-mediated oocyte maturation, P53 signaling pathway, cellular senescence, oocyte meiosis, human T-cell leukemia virus 1 infection, FoxO signaling pathway, ubiquitin-mediated proteolysis, viral carcinogenesis, and human immunodeficiency virus 1 infection were the top ten pathways enriched (Fig. 3a).

### Stemness enrichment analysis

**Fig. 4:**
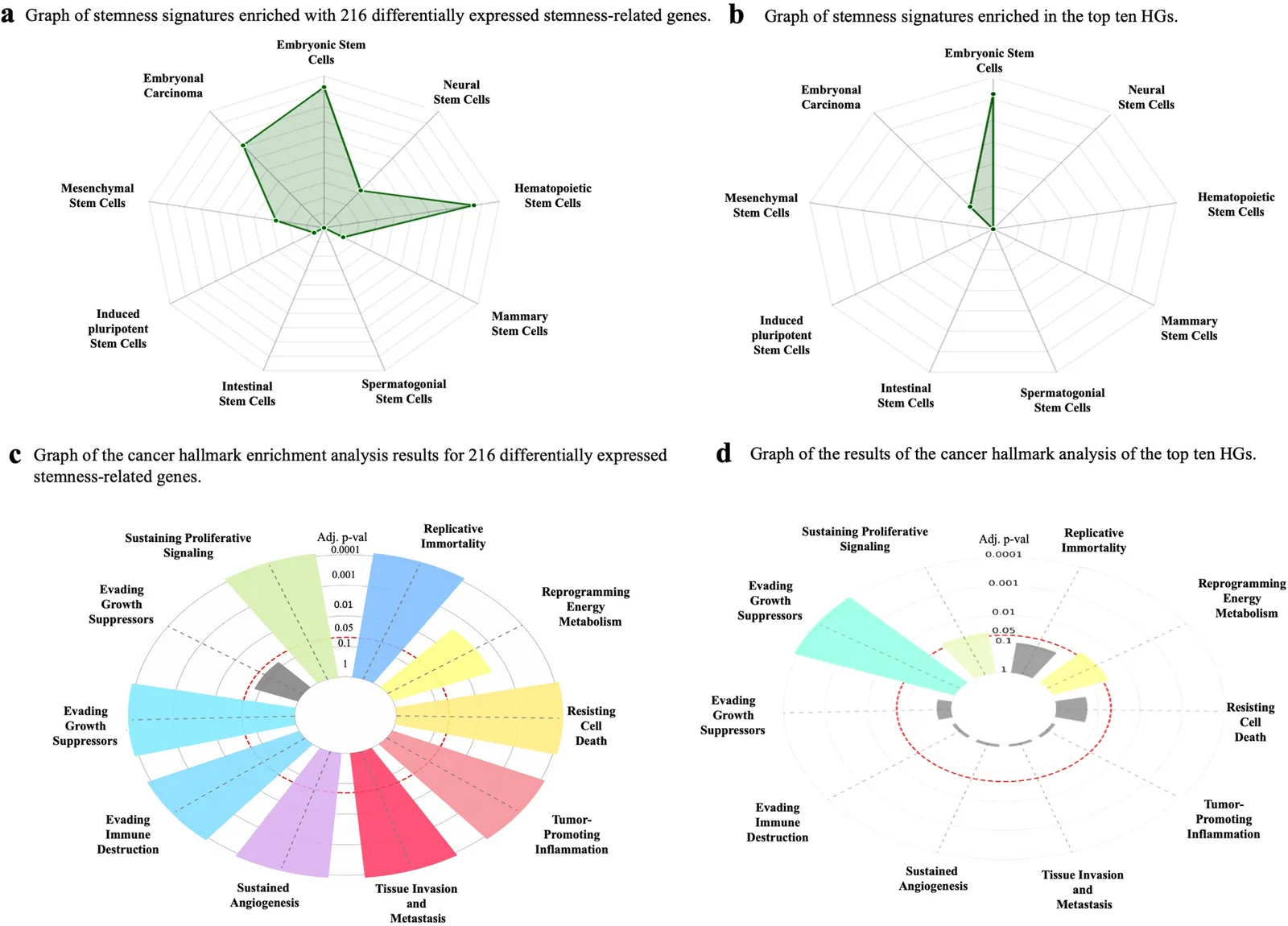
Stemness signature and cancer hallmark enrichment analysis. **a** Graph of stemness signatures enriched with 216 differentially expressed stemness-related genes. **b** Graph of stemness signatures enriched in the top ten HGs. **c** Graph of the cancer hallmark enrichment analysis results for 216 differentially expressed stemness-related genes. **d** Graph of the results of the cancer hallmark analysis of the top ten HGs

We next performed stemness signature enrichment analysis separately for 216 DEGs (Fig. 4a) and 10 HGs (Fig. 4b). An analysis of 216 stemness-related genes identified by StemChecker revealed significant enrichment of genes related to embryonic stem cells (70), hematopoietic stem cells (34), embryonal carcinoma (25), neural stem cells (8), mammary stem cells (7), mesenchymal stem cells (6), and induced pluripotent stem cells (Fig. 4a). Analysis of HGs via StemChecker revealed significant enrichment of genes related to embryonic stem cells and embryonic carcinoma (Fig. 4b). Data on stemness signatures and transcription factors associated with the DEGs are provided in Supplementary Tables S4, S5, and S6. The level of significance was estimated via hypergeometric tests and Bonferroni correction via comparisons against stemness signature genes and transcription factors curated manually via a literature search.

### Cancer hallmark enrichment analysis

Cancer hallmark enrichment analysis was carried out for stemness-related HGs via the Cancer hallmark enrichment analysis web server. This web server helps prioritize the cancer hallmarks in a given gene list by comparison against 6,763 cancer hallmark genes. We found a significant overlap of stemness-related HGs with those related to sustained proliferative signaling, genome instability, and energy metabolism reprogramming (Fig. 4c and d).

### HGs are overexpressed in CC samples

**Fig. 5:**
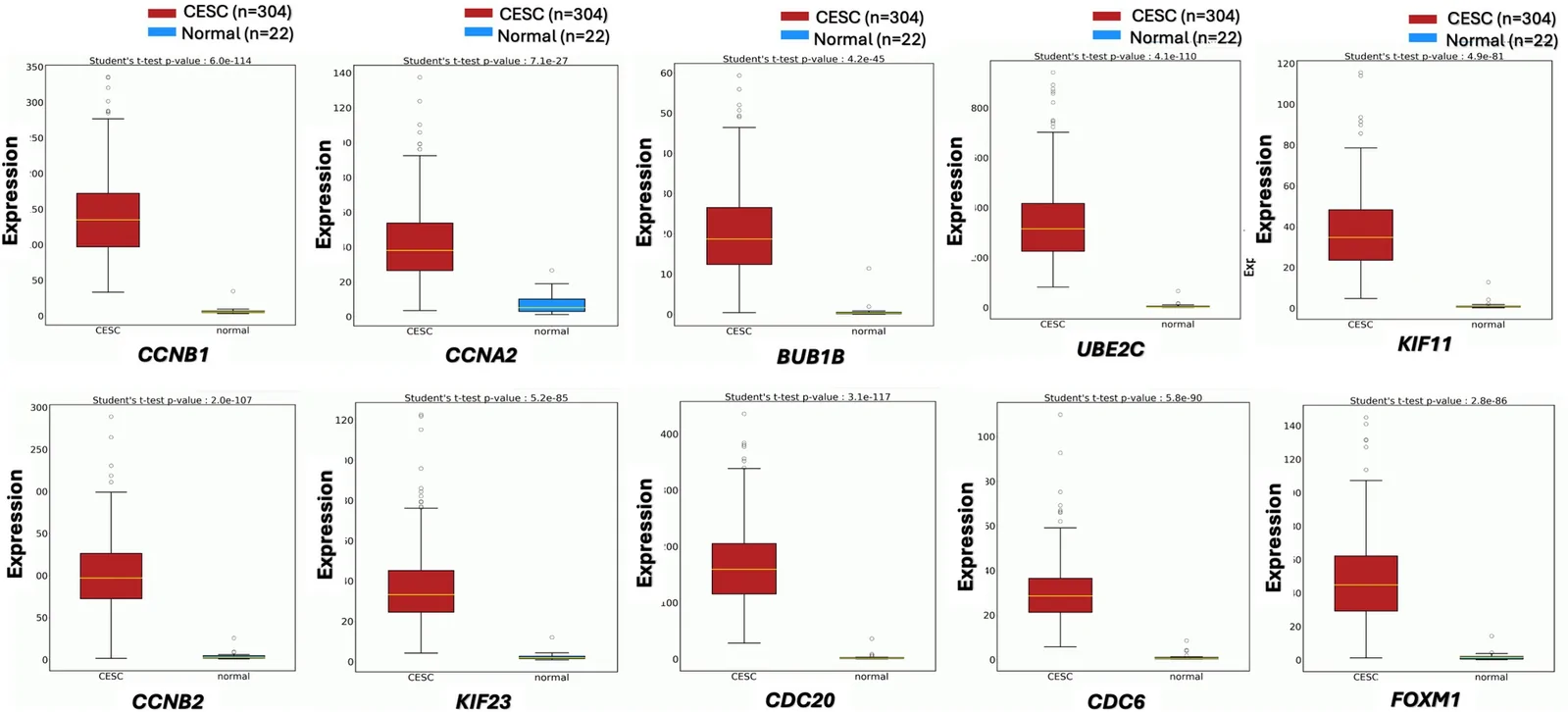
Relative expression analysis of HGs in normal and tumor samples. Representative expression analysis of the top ten HGs in the TCGA-CESC dataset via the OncoDB web server. All ten genes were significantly overexpressed in the TCGA-CESC dataset

We analyzed the expression of individual HGs in the TCGA-CESC dataset. Our analysis revealed significant overexpression of all the HGs in the tumor samples compared with the normal samples (Fig. 5).

### HG expression analysis (HPA IHC data)

**Fig. 6:**
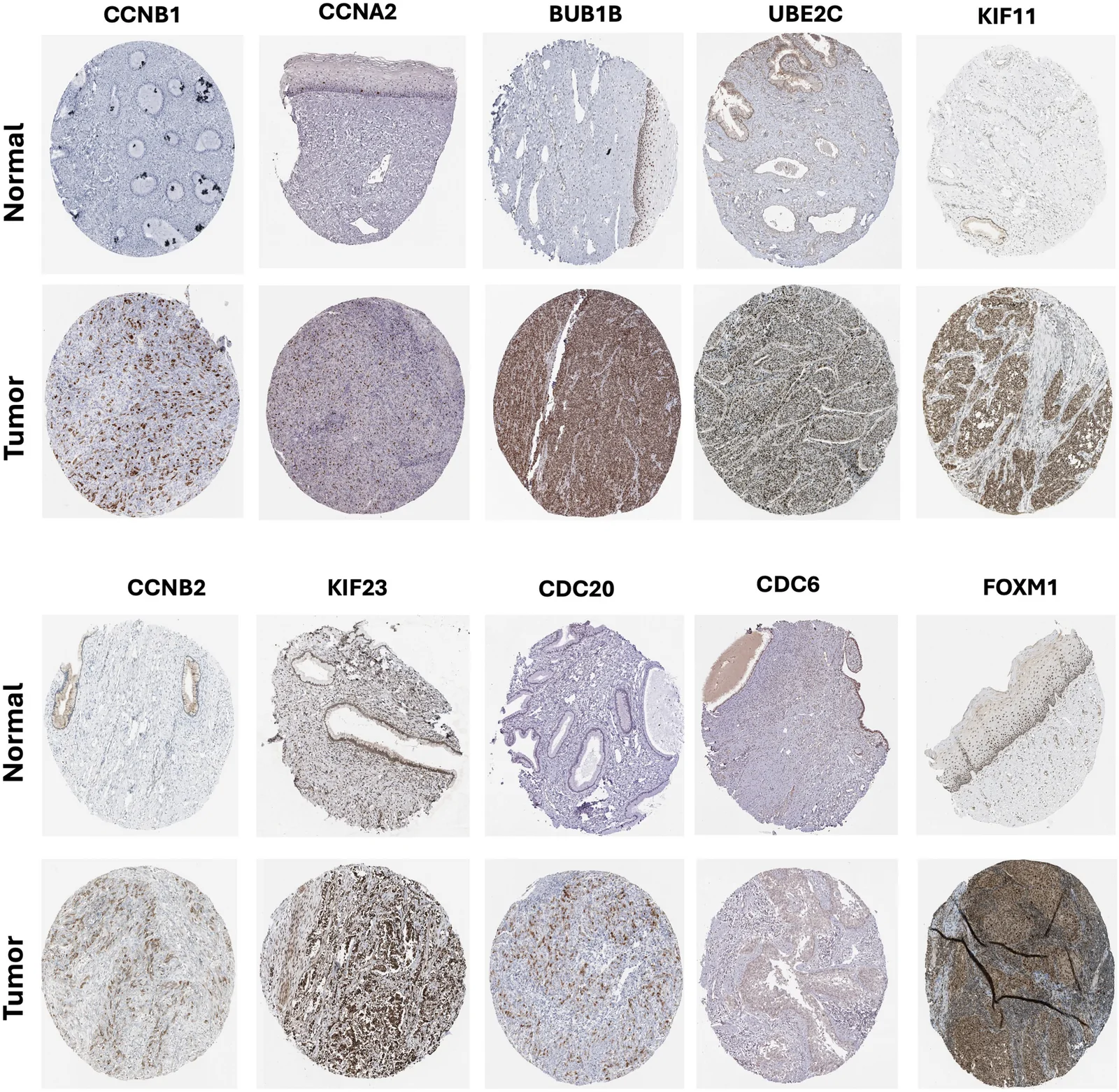
HPA analysis of the ten HGs in normal and cervical cancer samples. Representative immunohistochemistry (IHC) images depicting the protein expression of the ten HGs (*CCNB1*,* CCNA2*,* BUB1B*,* UBE2C*,* KIF11*,* CCNB2*,* KIF23*,* CDC20*,* CDC6*, and *FOXM1*) in normal cervical tissues and cervical cancer tissues obtained from the Human Protein Atlas (HPA) database. The images demonstrate differences in protein expression patterns between normal and tumor tissues

Analysis of the HPA IHC data revealed an increase in the expression of most HGs in CC samples compared with normal samples. In normal tissues, expression ranged from negative (CCNB1) to low (CCNB2 and FOXM1) and medium (CCNA2, CDC20, CDC6, KIF11, KIF23, and UBE2C) levels. However, CC samples presented high frequencies of medium-to-high protein expression. Furthermore, the expression of CDC6 was intermediate to high in 2 out of the 10 CC samples. CCNB1, CCNA2, and CCNB2 proteins were moderately to highly expressed in 8 out of 12 samples. The rest of the proteins were moderately to highly expressed in nearly all the samples, as determined by IHC (Fig. 6).

### Association of the GSVA score with tumor stage

GSVA across the TCGA-CESC dataset revealed enrichment of stemness-related HGs signatures in tumor tissues compared with normal controls. The GSVA score of the tumor samples ranged from 0.23 to 0.71 (Mean = 0.559 ± 0.077), with approximately 75% of the tumor samples having a score above 0.5. However, the mean value of the GSVA score in normal samples was − 0.22. Our analysis revealed the widespread abundance of stemness pathways in tumor samples. The mean GSVA score across clinical stages was constant and consistently high. The mean GSVA scores for Stage 1 (*n* = 162), Stage II (*n* = 69), Stage III (*n* = 46), and Stage IV (*n* = 22) were 0.55, 0.57, 0.56, and 0.59, respectively. GSVA suggested that stemness pathway enrichment may be a consistent feature of CC from early to advanced stages (Supplementary Fig. S3a) and Supplementary Table S7).

### Testing the prognostic value of stemness-related HGs in CC

**Fig. 7:**
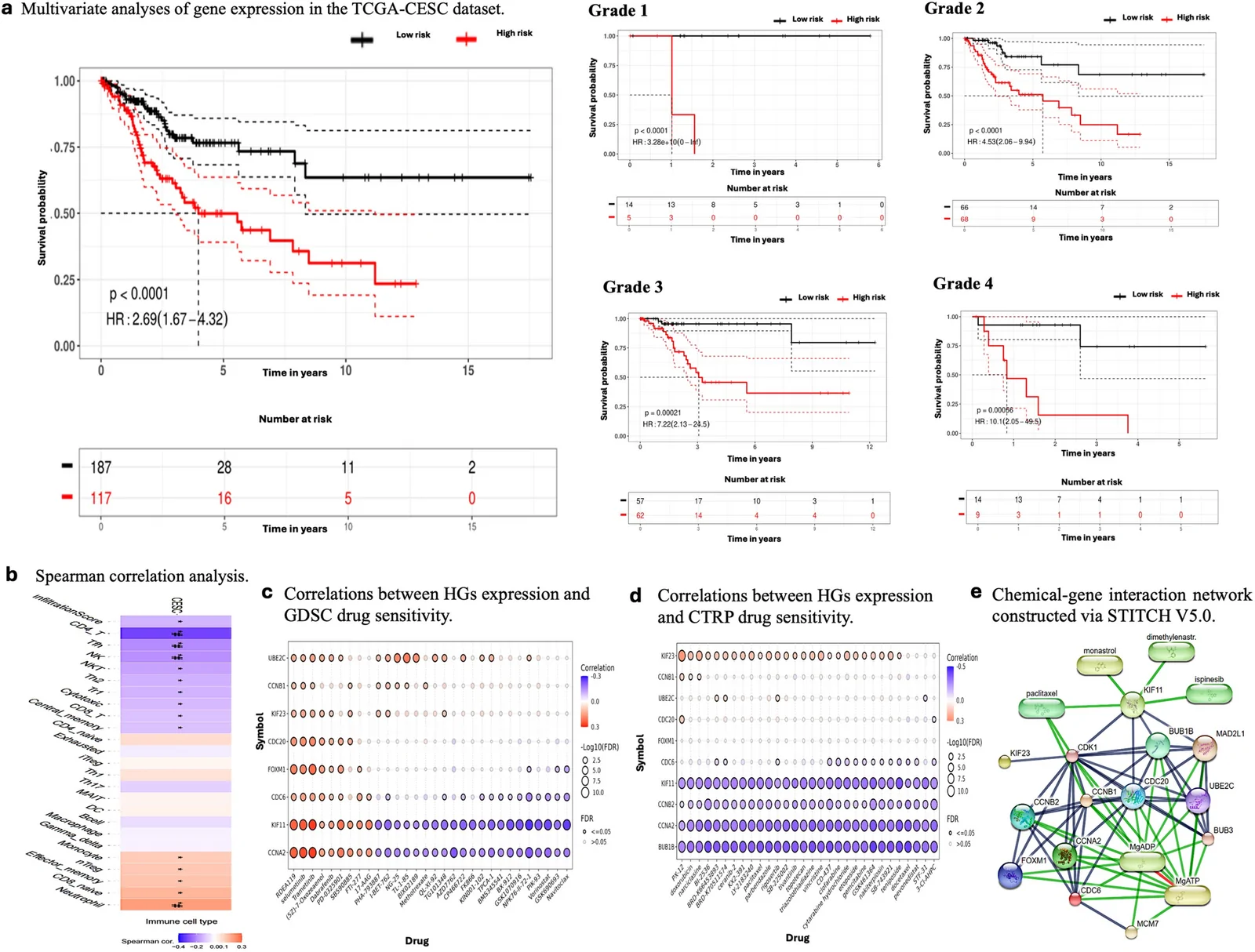
Analysis of prognosis, immune cell abundance, and drug‒gene interactions. **a** Multivariate analyses of gene expression in the TCGA-CESC dataset. The risk score= (0.373 *BUB1B) +(0.565 *CCNA2) +(-0.469 *CCNB1) +(-0.176 *CCNB2) +(0.352 *CDC20) +(-0.177 *CDC6) +(0.0765 *FOXM1) +(-0.559 *KIF11) +(0.0619 *KIF23) +(-0.114 *UBE2C) was constructed via the ToPP tool. *BUB1B*, *CCNA2*, *CDC20*, *FOXM1*, and *KIF23* emerged as risk factors for poor overall survival. The higher the risk score is, the worse the prognosis. All patients were divided into high- and low-risk groups according to the median risk score. Additionally, *KIF11* emerged as an independent prognostic factor for overall survival. Grade 1: Multivariate analyses for gene expression in the TCGA-CESC cohort. The risk score=(30.9 *BUB1B)+(-25.8 *CCNA2)+(47.8 *CCNB1)+(-46.7 *CCNB2)+(12.4 *CDC20)+(-21.1 *CDC6)+(-26.1 *FOXM1)+(-40.4 *KIF11)+(18 *KIF23)+(64 *UBE2C). Among them, *BUB1B*, *CCNB1*, *CDC20*, *KIF23*, and *UBE2C* are risk factors. Grade 2: Multivariate analyses for gene expression in the TCGA-CESC cohort. The risk score=(0.491 *BUB1B)+(0.448 *CCNA2)+(-0.376 *CCNB1)+(-0.19 *CCNB2)+(0.703 *CDC20)+(-0.234 *CDC6)+(0.332 *FOXM1)+(-0.833 *KIF11)+(0.000853 *KIF23)+(-0.458 *UBE2C). Among them, *BUB1B*, *CCNA2*, *CDC20*, *FOXM1*, and *KIF23* were risk factors. Grade 3: Multivariate analyses for gene expression in the TCGA-CESC cohort. The risk score=(0.878 *BUB1B)+(0.785 *CCNA2)+(-0.519 *CCNB1)+(-0.132 *CCNB2)+(-0.476 *CDC20)+(0.259 *CDC6)+(-0.34 *FOXM1)+(-0.696 *KIF11)+(-0.348 *KIF23)+(0.609 *UBE2C). Among them, *BUB1B*, *CCNA2*, *CDC6*, and *UBE2C* are risk factors. Grade GX: Multivariate analyses of gene expression in the TCGA-CESC cohort. The risk score=(3.48 *BUB1B)+(-2.13 *CCNA2)+(1.09 *CCNB1)+(-4.14 *CCNB2)+(-2.81 *CDC20)+(4.82 *CDC6)+(-0.714 *FOXM1)+(-1.34 *KIF11)+(0.354 *KIF23)+(3.49 *UBE2C). Among them, *BUB1B*, *CCNB1*, *CDC6*, *KIF23*, and *UBE2C* are risk factors. **b** Spearman correlation analysis between HGs expression and the infiltration of 24 different immune cells. **c** Correlations between HGs expression and GDSC drug sensitivity. **d** Correlations between HGs expression and CTRP drug sensitivity. A positive correlation indicates that increased gene expression may lead to drug resistance. A negative correlation indicates that increased gene expression may increase drug sensitivity. **e** Chemical‒gene interaction network constructed via STITCH V5.0

The prognostic value of 10 stemness-related HGs in predicting CC patient survival was determined by comparing their expression levels with patient survival outcomes via a tumor online prognostic analysis platform. The patients were categorized into high-expression and low-expression groups using the median gene expression value as the cutoff. The median threshold was selected because it is a commonly adopted and statistically robust approach in gene expression survival studies. Median-based analysis generated balanced comparison groups while reducing the influence of outliers and skewed expression distributions. Furthermore, the ToPP tool used in this study employs the median expression value as its default cutoff for K-M survival analysis. Multivariate analysis was carried out by applying a Cox regression model. A log-rank test was carried out to estimate the differences in survival between the high-expression and low-expression groups. Among the 10 HGs, *BUB1B*,* CCNA2*,* CDC20*,* FOXM1*, and *KIF23* were considered risk factors that may contribute to a poor prognosis (Fig. 7a). Multivariate analysis also revealed that *KIF11* is an independent prognostic factor in CC. Supplementary Fig. S4 shows the K-M plot for survival analysis of individual genes in the TCGA-CESC dataset and the American Joint Committee on Cancer (AJCC) pathologic tumor staging.

### Grade-specific multivariate risk models

Multivariate Cox regression analysis was performed to evaluate the prognostic impact of ten genes on overall survival in TCGA-CESC patients stratified by tumor grade. *BUB1B*,* CCNB1*,* CDC20*,* KIF23*, and *UBE2C* were identified as risk factors, with higher expression associated with decreased survival in patients with grade 1 tumors. High expression of *BUB1B*,* CCNA2*,* CDC20*,* FOXM1*, and *KIF23* was identified as a risk factor for overall survival in patients with Grade 2 tumors. In Grade 3 tumors, genes such as *BUB1B*,* CCNA2*,* CDC6*, and *UBE2C* emerged as risk factors. In Grade GX (unknown grade), *BUB1B*,* CCNB1*,* CDC6*,* KIF23*, and *UBE2C* emerged as risk factors. Collectively, our results suggest that *BUB1B* and *UBE2C* are consistently risk factors across all four grade subgroups (Fig. 7a).

### Stemness-related HGs expression is correlated with immune infiltration

We performed an in silico analysis to identify the correlation between the expression of stemness-related HGs and immune infiltration via the GSCA web server. The GSVA score for HGs calculated via the ImmuCellAI algorithm suggested a significant association between the expression of stemness-related HGs and that of specific immune cells (Fig. 7b; P value ≤ 0.05; FDR ≤ 0.05). Spearman correlation analysis revealed significant negative associations between CD4 + T, Tfh, NK, NKT, Th2, Tr1, cytotoxic, CD8 + T, and central memory cell levels and the expression of stemness HGs (Fig. 7b). In contrast, monocyte, nTreg, effector and memory T cell, CD8 naive and neutrophil counts were significantly positively associated with the expression of stemness HGs (Fig. 7b).

### Stemness-related HGs expression is correlated with the expression of small molecules and drugs

Aberrant expression of stemness-related HGs affects prognosis and immune infiltration. HGs may represent potential therapeutic targets for further investigation. Therefore, we performed in silico analysis via the GSCA web tool to identify small molecules and drugs that can target HGs. A correlation map of the top 30 drugs/chemicals associated with stemness-related HGs expression is shown in Fig. 7c and d. The associations between different chemicals and HGs are shown in Fig. 7e. Drug sensitivity analysis via the GDSC identified 2008 chemicals/drugs that target stemness-related HGs. Further analysis of 2008 drugs/chemicals revealed 251 unique compounds that target 10 stemness-related HGs (Supplementary Table S8). Similarly, CTRP sensitivity analysis revealed 4810 drugs/chemicals that target 10 stemness-related HGs (Supplementary Table S9). Further filtering of the 4810 drugs/chemicals resulted in 481 unique drugs/chemicals. In addition, filtering 251 and 481 unique drugs obtained through the GDSC and CTRP tools, respectively, yielded 661 unique drugs/chemicals to target stemness-related HGs (Supplementary Table S10).

### *FOXM1* and *KIF11* are overexpressed in CC tissues and cell lines

**Fig. 8:**
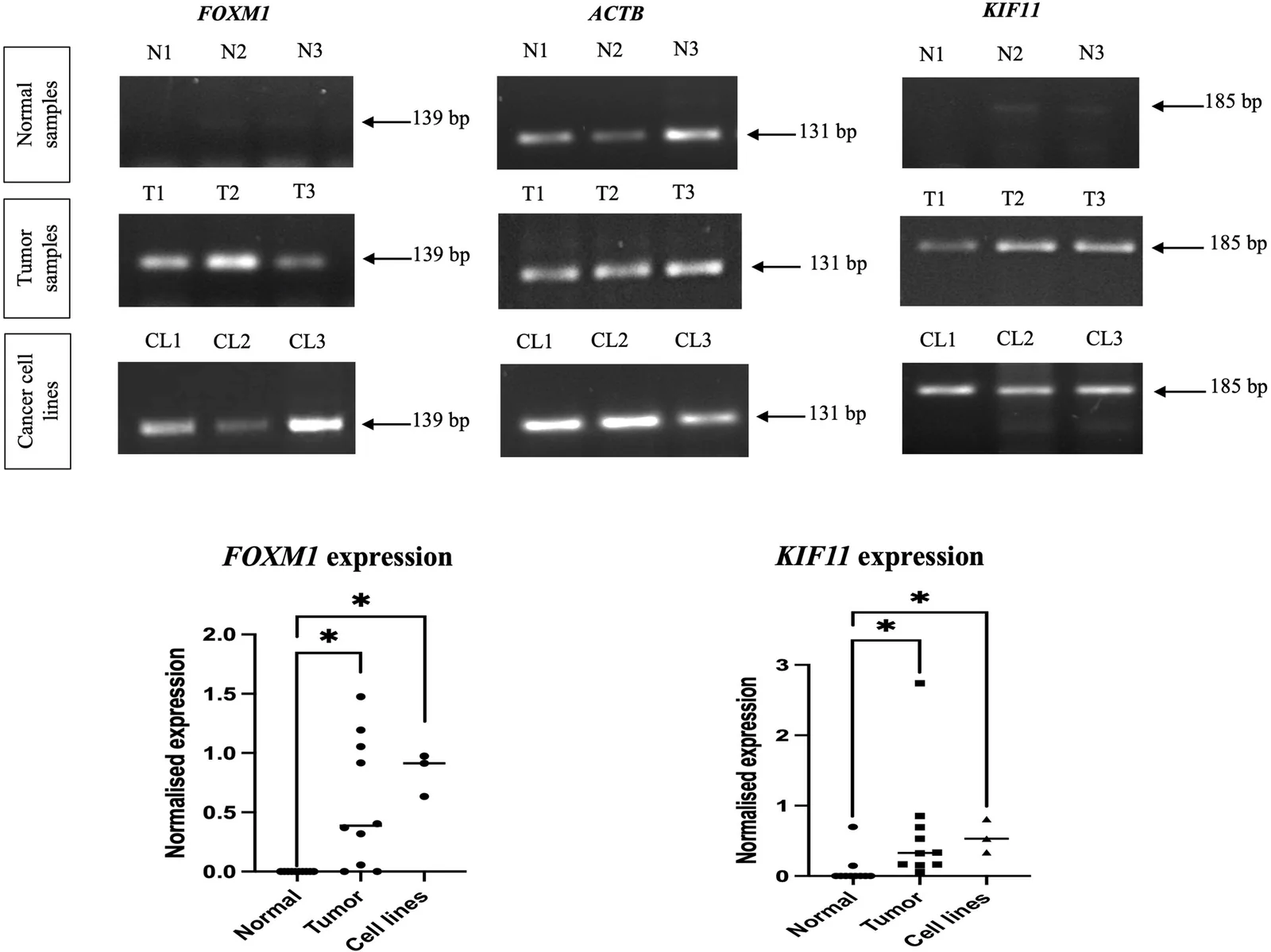
Validation of the expression of the two HGs by RT‒PCR. Representative RT‒PCR gel images showing *FOXM1* (139 bp), *ACTB* (131 bp) and *KIF11* (185 bp) amplification in normal cervical tissue (N1–N3), tumor tissue (T1–T3), and CC cell lines (CL1–CL3). Scatter plots showing normalized *FOXM1* and *KIF11* expression across the three groups. The expression values were normalized to those of *ACTB*. Statistical significance was determined via the Kruskal–Wallis test followed by Dunn’s multiple comparisons test. Compared with normal tissue, tumor and cell line samples presented significantly greater expression of *FOXM1* and *KIF11* (*p < 0.05)

To validate the stemness-associated HGs identified through in silico analysis, the expression of *FOXM1* and *KIF11* was experimentally assessed via semiquantitative RT‒PCR in normal cervical tissues, CC tissues, and CC cell lines. RT‒PCR analysis revealed a marked increase in the expression of *FOXM1* and *KIF11* in tumor tissues and CC cell lines compared to normal cervical tissues, whereas *ACTB* was stably expressed across all samples (Fig. 8). Densitometric quantification was performed via ImageJ software, and the expression levels were normalized to those of *ACTB*. Quantitative analysis revealed significant upregulation of *FOXM1* and *KIF11* in CC tissues and cell lines compared with normal samples (Fig. 8). Statistical analysis via the Kruskal–Wallis test followed by Dunn’s multiple comparisons test confirmed that the observed differences were statistically significant (*p < 0.05). These results were consistent with the in silico findings, confirming the overexpression of *FOXM1* and *KIF11* in CC. Additionally, we performed differential gene expression analysis of the ten HGs using three different GEO datasets (GSE6791, GSE9750, and GSE39001), and we observed that the expression of the HGs was consistently upregulated in CC tissues compared with that in normal tissues, with varying degrees of change (Supplementary Fig. S3 b).

## Discussion

CSCs promote growth, metastasis, recurrence, and therapeutic resistance in CC [3]. Owing to its tumor-promoting properties and ability to influence treatment outcomes, identifying the genes and pathways contributing to the stemness of CC is important. In this study, we identified stemness-related genes in CC via analysis of the TCGA-CESC dataset via multiple bioinformatics tools. In this study, stemness-associated HGs in CC were characterized, and their biological, prognostic, immune, and therapeutic relevance were evaluated through integrated bioinformatics analyses and experimental validation. Finally, to substantiate our in silico findings, we performed RT‒PCR for two of the HGs, *FOXM1* and *KIF11*. The data presented may have clinical utility, as these data were generated from either genome-wide studies from CC patients or CC cell lines. We first conducted a literature search to identify DEGs related to stemness. We compared the stemness-related genes identified by a literature search with DEGs in the TCGA-CESC dataset. We constructed and analyzed the PPIN to identify the top ten highly connected genes. Although the 10 HGs identified in the current study (*CCNB1*,* CCNA2*,* BUB1B*,* UBE2C*,* KIF11*,* CCNB2*,* KIF23*,* CDC20*,* CDC6*,* and FOXM1*) were previously reported to be upregulated in CC [31–33], their role in regulating stemness properties in CC is very limited. HPA-based IHC analysis suggested the general upregulation of HGs in CC samples. Interestingly, these proteins are linked to cell cycle regulation, mitotic progression, chromosome segregation, and cell proliferation, suggesting the potential for dysregulation of genomic stability pathways in cervical cancer. Low or moderate expression levels in normal samples and frequently high to medium expression levels in tumor samples highlight the clinical relevance of these proteins in CC.

Among these 10 HGs, only *FOXM1* has been previously studied for its stemness-related roles in CC [34–35]. GO and pathway analyses revealed that the ten HGs were primarily associated with cell division and cell cycle progression. A dysregulated cell cycle is often observed in CC, which promotes cell proliferation and growth. Among these genes, *CCNB1*, a member of the cyclin family, plays a crucial role in controlling the G2/M phase transition during the cell cycle [36]. A previous in silico investigation revealed the overexpression of *CCNB1* and identified it as one of the HGs in CC [37]. *CCNB1* overexpression is associated with the CRNDE/miR-183/*CCNB1* axis and CC progression [38]. *CCNA2* belongs to the cyclin family and is a regulator of cyclin-dependent kinases [32]. *CCNA2* was previously reported to be upregulated and to promote the G1 to S-phase transition [39]. *BUB1B* is a kinase localized to the kinetochore that is crucial for controlling spindle checkpoint functions to ensure the correct segregation of chromosomes [40]. One study revealed the upregulation of *BUB1B* in CIN 2/3 patients [32]. Previous in silico studies revealed *BUB1B* upregulation and its prognostic value in patients with CC. *UBE2C* was another hub gene whose expression was significantly upregulated in our analysis. *UBE2C* encodes an E2 ubiquitin-conjugating enzyme, which is a component of the anaphase-promoting complex and is needed for controlling cell cycle [41]. *UBE2C* overexpression is reported to impact chromosome segregation and promote cell cycle progression [41]. *UBE2C* is a highly upregulated gene in CC tissues that promotes cell proliferation in vitro and regulates the mTOR/PI3K/AKT axis in vivo [33]. Functional studies have suggested that *UBE2C* is a promoter of EMT [42]. Furthermore, exposure to dietary compounds such as flavonoids inhibited CC invasion by downregulating *UBE2S*-mediated EMT [42]. The downregulation of miR-525-5p expression is connected to metastasis and anoikis resistance in CC via the activation of the *UBE2C/ZEB1*/2 axis [43].

The present study revealed that *KIF11* was upregulated in CC tissues compared with normal samples. Our experimental validation revealed similar results, with *KIF11* being overexpressed in CC tissue samples and cell lines compared with normal samples. *KIF11*, a chromosome instability gene critical for the positioning of chromosomes, separation of the centrosome and establishment of bipolar spindles during mitosis, has already been shown to be upregulated in various cancers, leading to their progression [44]. Previous in silico studies have shown the upregulation of *KIF11* in CC tissue samples [45]. Another study reported that *KIF11* is a potential prognostic factor in CC [46]. However, the mechanism by which *KIF11* promotes CC is not fully understood. *CCNB2* is a member of the cyclin family that is important for controlling the G2/M transition during mitosis. We report that *CCNB2* upregulation occurs in CC tissue samples. Both *CCNB1* and *CCNB2* bind to CDK1 to promote the G2/M transition. A microarray-based analysis revealed that compared with normal samples, *CCNB2* is among the top 6 genes that are highly overexpressed in HPV-16-positive CC samples. *KIF23* was another upregulated hub gene in our study. It is a kinesin superfamily member critical for cytokinesis during mitosis. Studies on the overexpression and knockdown of *KIF23* have shown that it promotes CC by targeting the activation of pyroptosis [47]. *CDC20* is an oncogene that promotes the growth of cancer cells by targeting multiple signaling pathways [48]. An earlier study revealed higher levels of *CDC20* expression in CC cells than in control cells. The upregulation of *CDC20* in high-grade squamous intraepithelial lesions and cervical squamous cell carcinoma tissue samples compared with normal samples has been previously reported [49]. Given that *CDC20* is overexpressed in the early stages of CC, it may be a useful candidate for early CC diagnosis. One study reported that *CDC6* is preferentially expressed in high-grade lesions and invasive squamous cell carcinomas of cervical samples [50]. Functional studies have suggested that *CDC6* has oncogenic functions in CC. The same study reported the role of *CDC6* in the E7-induced bypass of G1 checkpoints under hypoxic conditions [51]. Another study revealed the upregulation of *CDC6* in HPV-16 E7-overexpressing primary human keratinocyte cells [51]. Collectively, these studies indicate the potential role of HPV-encoded E7 in promoting cell cycle progression via the upregulation of *CDC6*. We further validated the expression levels of *FOXM1* and observed that the expression of this gene was significantly higher in CC tissue samples and cell lines than in normal samples. It is an overexpressed transcription factor that promotes proliferation and anchorage-independent growth in vitro. The interaction of *FOXM1* with *ABCC5* contributes to paclitaxel resistance in CC [52]. *FOXM1* is upregulated in early-stage CC patients and is associated with poor survival. Functional investigations revealed that *FOXM1* can promote migration and invasion and increase MMP2 and MMP1 levels in CC cells [53]. These findings suggest that the upregulation of stemness-related HGs may be associated with cell cycle progression and enhanced proliferation in CC cells.

Our analysis revealed a difference in GSVA scores between CESC tumor and normal samples, suggesting the prominent role of these HGs and the activation of stemness-related pathways in the majority of CC samples. The wide range of scores (0.235–0.715) may indicate molecular heterogeneity within the CC. Stage-stratified analysis revealed that the mean GSVA score was consistent from Stage I through Stage IV, with a modest increase in Stage IV. These findings suggest that the activation of the stemness pathway may occur early and persistently in CC than during the advanced stage of cancer. However, the use of only a small number of normal samples in the TCGA-CESC dataset is a limitation of the GSVA performed in the current study. Furthermore, the GSVA score reflects pathway enrichment rather than a direct estimation of the frequency of the cancer stem cell population.

Our findings are in line with several recent bioinformatics studies conducted in the Indian context. For instance, Shukla et al. (2023) [54] identified key HGs associated with the miR-200b/429 network in CC and reported their potential in prognosis and clinical management. Similarly, Vaghasia et al. (2022) [37] performed interactive bioinformatics analysis to screen HGs and revealed *CDK1* as a potential therapeutic target through molecular docking. More recently, Shekhawat et al. (2025) [55] and Chand et al. (2025) [56] highlighted overlapping cell cycle-related HGs (including *CCNB2*, *CDC20*, and *AURKA*) as potential diagnostic and prognostic biomarkers. While these studies focused primarily on general differential expression or specific pathways, the present work uniquely integrates stemness-associated gene selection with multialgorithm HGs identification, extensive drug sensitivity analysis, immune infiltration profiling and experimental validation, thereby providing a more comprehensive stemness-focused perspective.

Prognostic markers are clinical indicators employed by oncologists to determine a patient’s likelihood of disease recurrence following initial treatment. These markers help clinicians categorize patients into risk categories and decide treatment strategies. Hence, we investigated the prognostic utility of HGs in CC and created a prognostic model on the basis of HGs expression. Cox regression analysis and log-rank tests suggested that patients with high expression had poor overall survival. Multivariate analysis-based prognostic model construction revealed that the overexpression of *BUB1B*,* CCNA2*,* CDC20*,* FOXM1*, and *KIF23* could be considered risk factors for poor prognosis and is associated with decreased overall survival.

In the present study, we identified a grade-specific prognostic signature for 10 HGs. We showed that the risk status of several genes varies substantially across tumor grades. Our analysis supports the importance of clinical stratification of samples for survival modeling. *BUB1B* and *UBE2C* were consistently risk factors across all tumor grades, suggesting that they may drive poor prognosis regardless of tumor differentiation. Despite this, the small sample size and lack of external validation are some of the limitations of the present study. Further validation in independent cohorts and functional studies is needed to understand the mechanistic basis of these genes in CC.

By performing stemness enrichment analysis, we showed that the 10 HGs are direct targets of stemness-inducing genes and transcription factors. HGs were enriched in gene signatures associated with embryonal carcinoma and embryonic stem cells. Genes and transcription factors that are enriched in embryonic stem cells have also been found in poorly differentiated cancers. Although several of these genes have been implicated in CC, further mechanistic and clinical studies are needed to elucidate their precise roles and interactions in disease progression. Our analysis revealed the coexpression of several of these genes in CC, suggesting their potential cooperative involvement in disease progression. Thus, in-depth mechanistic studies are needed to understand the molecular mechanisms affected by coexpressed genes and their contribution to CC progression.

Another key finding of our study is the link between the upregulation of HGs and immune cell infiltration. The TME plays key roles in tumor growth, proliferation, invasion, migration, metastasis, treatment resistance and stemness maintenance [57]. The TME includes immune cells, cytokines and chemokines, blood cells, stomal cells, fibroblasts, and the extracellular matrix. We analyzed the associations of stemness-related HGs expression with the abundance of immune cells via the TCGA-CESC dataset in the GSCA web server. We found a significant negative association between HGs upregulation and CD4 + T, Tfh, NK, NKT, Th2, Tr1, cytotoxic, and CD8 + T and central memory cell abundances and positive associations with monocyte, nTreg, effector memory, and CD8 + naive and neutrophil abundances. HPV infection and compromised immunity are the major risk factors for the development and progression of CC. Our in silico investigation suggested that the overexpression of HGs can influence the abundance of different immune cells. Furthermore, changes in the abundance of these immune cells in tumors are common in CC. For example, the development of CC is associated with the suppression of the CD4 + T-cell response to HPV [58]. Similarly, Tfh cells support the ability of B cells to produce a strong antibody response, and their presence in tumor tissue is correlated with better clinical outcomes in many solid tumors [59]. NK cells are lymphocytes that can identify and eliminate cancer cells. The abundance of natural killer (NK) cells within tumors is correlated with good prognosis in many cancers [60]. These associations indicate a potential relationship between stemness-related HGs expression and the immune microenvironment, which warrants further investigation in the context of immunotherapy.

The development of therapeutic resistance is a major contributor to high mortality in various cancers, including CC. Drug sensitivity analysis revealed several candidate compounds and repurposable agents targeting the HGs. Experimental and clinical validation are needed to identify the most robust candidate. Among the ten HGs, *FOXM1* and *KIF11* are particularly promising therapeutic targets. *FOXM1* is a transcription factor that is overexpressed in multiple cancers, and its small-molecule inhibitor FDI-6 has shown preclinical efficacy. *KIF11* (also known as Eg5) is a mitotic kinesin with several inhibitors in clinical trials (ispinesib, SB-743921) for solid tumors. Additionally, 661 unique drugs targeting these HGs, such as paclitaxel (targets *KIF11* indirectly), cisplatin, and vorinostat, have been approved by the FDA. FDA-approved drugs identified through sensitivity analysis may represent candidates for future preclinical evaluation.

### Strengths and limitations

This study has several strengths: (i) comprehensive integration of multiple public databases; (ii) a multialgorithm approach for HGs identification; (iii) cross-validation using an independent GEO dataset; (iv) experimental validation of two HGs; and (v) drug repurposing analysis with molecular docking. However, several limitations must be acknowledged. First, clinical covariates (age, tumor stage, HPV status, and treatment history) were not included because of incomplete annotation in the selected datasets, which may introduce confounding. Second, compared with the tumor samples (n=304), the normal samples (*n* = 22) were relatively small. Third, despite identifying stemness-associated genes through literature mining, CellMarker 2.0, and overlapping with DEGs, we did not use advanced stemness scoring systems such as mRNAsi-based analyses. This method could provide more insights into cancer stemness properties and further strengthen the association between the identified HGs and stemness characteristics. Fourth, the RT‒PCR validation was semiquantitative and limited to two HGs. Fifth, no functional experiments were performed to confirm the stemness-related roles of these genes. Sixth, drug predictions are entirely in silico and require in vitro and in vivo validation. We recommend that these limitations be addressed in future studies.

## Conclusion

We identified 216 stemness-related genes and 10 HGs that were differentially expressed between CC and normal samples and validated two of the HGs in clinical samples. These HGs are associated with stemness signatures and may be linked to biological processes involved in CC progression. The upregulation of HGs may affect the overall survival of CC patients. These genes may represent potential therapeutic targets and provide a foundation for future studies exploring stemness-related mechanisms in CC.

## Supplementary Information

Supplementary Material 1. Supplementary Figure S1: Methylation analysis of HGs. Among the 10 HGs evaluated, CCNB1 and CCNA2 were significantly differentially methylated between normal and tumor samples. The box plot represents the beta values in normal and tumor samples. We used the UALCAN web server for analysis.

Supplementary Material 2. Supplementary Figure S2: Analysis of HGs for genomic changes. a) Oncoprint analysis of 10 HGs via the cBioPortal web server. Missense mutations, splice mutations, truncating mutations, amplifications, and deletions have been reported for few genes. Among the ten HGs, KIF11 presented the greatest number of alterations. b) Gene map showing the distribution of various types of genomic changes in the TCGA-CESC dataset.

Supplementary Material 3. Supplementary Figure S3: Independent validation of the HGs in external GEO datasets and their association with disease stage. a) Box plot showing the GSVA enrichment score of the HGs signature in different clinical stages (I–IV) of cervical squamous cell carcinoma. b) Bar graph showing consistent upregulation of all ten HGs across three GEO datasets (GSE6791, GSE9750, and GSE39001). The height of the bars represents the log2-fold change (log2FC) in cervical cancer tissues compared with normal controls.

Supplementary Material 4. Supplementary Figure S4: Overall survival analysis. Kaplan‒Meier survival analysis for individual HGs in the TCGA-CESC dataset and the AJCC pathologic tumor staging system. Among the genes analyzed, CCNA2 and KIF11 expression significantly affected the overall survival of CC patients.

Supplementary Material 5. Supplementary Table S1: List of differentially expressed genes in the TCGA-CESC dataset. Supplementary Table S2: List of differentially expressed stemness-related genes in cervical cancer. Supplementary Table S3: Common genes between the TCGA-CESC dataset and stemness-related genes. Supplementary Table S4: List of overlapping genes based on stem cell type analyzed using StemChecker. Supplementary Table S5: List of overlapping genes based on stemness signatures analyzed in StemChecker. Supplementary Table S6: List of DEGs regulated by transcription factors analyzed using StemChecker. Supplementary Table S7: GSVA Stemness Scores of Cervical Cancer Samples from the TCGA-CESC Dataset. Supplementary Table S8: Drug sensitivity analysis of stemness-related hub genes in cervical cancer using the GDSC tool. Supplementary Table S9: Drug sensitivity analysis of stemness-related hub genes in cervical cancer using the CTRP tool. Supplementary Table S10: List of unique drugs/chemicals that target stemness-related hub genes.

## Data Availability

No datasets were generated or analysed during the current study.
